# The pandemic of coronavirus disease 2019 (COVID-19): The good, the bad and the ugly!

**DOI:** 10.1017/ice.2021.46

**Published:** 2021-02-04

**Authors:** Syed A. Sattar

**Affiliations:** Faculty of Medicine, University of Ottawa, Ottawa, Ontario, Canada

*To the Editor—*We will remember 2020 as the ‘Year of the Pandemic’ for the immense and ongoing loss of life, the unprecedented economic disruptions, and the persistent negative impacts on our social interactions it has wrought. At the start, our otherwise impressive advances in medicine of the past several decades simply could not match this wily enemy. Very soon though, we mobilized our ingenuity, determination, and technological prowess to find several vaccines and drugs to counter severe acute respiratory coronavirus virus 2 (SARS-CoV-2), the cause of coronavirus disease 2019 (COVID-19).^1^ The response has been impressive and heartening.

Vaccines and drugs aside, the pandemic reinforces the significance of the basic principles of infection prevention and control (IPAC) against a respiratory pathogen. IPAC, based mainly on personal and environmental hygiene, is generic and cost-effective and is not pathogenic specific, as shown by a decrease in the number of cases of influenza, for example, while fighting the current pandemic.^2^

Doubtless, we will beat the virus. However, I am concerned with the reluctance of many otherwise qualified and influential persons to recognize the new virus as a health threat despite the never-ending news of thousands dying daily along with the worldwide social and economic disruptions. Critical thinking aside, the socially irresponsible behavior of the ‘nay-sayers’ placesthemselves and countless others at the mercy of the virus! Such behavior is especially puzzling in the ‘developed’ regions with unlimited access to *credible* sources of news. Perhaps, that is the soft underbelly of the beast, allowing certain nefarious elements to use their bully pulpit to call the pandemic a ‘hoax’ or a conspiracy. Even now, the much needed vaccination is being tainted by accusations of microchip implantations!

The initial and tardy response of global public health agencies to the pandemic was not altogether surprising when faced with a brand new virus, which has indeed proven to behave differently from other respiratory coronaviruses in particular.^3^ Its lack of seasonality is particularly striking and puzzling. Based on the known predilection of enveloped viruses in general to fair better at low-to-medium levels (30%–50%) of relative humidity, a drop in the number of COVID-19 cases was expected with the seasonal rise in relative humidity, but that did not happen. Is it due to some unique feature of the virus or the fact that, as a ‘new’ virus, it has a ‘virgin’ or naïve global population to infect without any constraints of seasonality?

The year 2021 will be remembered as the ‘Year of the COVAX’ for the bold plans already underway to immunize much of the world population against SARS-CoV-2. Of course, the success of this plan will depend on the proportion of those willing to be vaccinated despite the baseless fearmongering against vaccine safety. Although it would be imprudent to predict the outcome of the mass vaccination on virus spread, one would hope for its success in breaking the back of the pandemic. With that anticipated positive outcome, those who deserve credit are all the frontline workers, researchers for the rapid vaccine development and testing, regulators for approving vaccine use in record time, and officials for keeping the public informed on the pandemic and measures to counter it.

Despite the phenomenal advances in virology, we do not yet understand how respiratory viruses spread in nature. This applies not only to SARS CoV-2 but also to human influenza viruses,^4^ which have been around for centuries. It took the joint efforts of many for the World Health Organization to rethink its initial position on the aerosol spread of COVID-19.^5^

Based on our successes against smallpox^6^ and poliomyelitis,^7^ we shall beat the pandemic of COVID-19 together in a year or two! The lessons from the pandemic should also prepare us better against likely new pathogens to come. Detractors aside, we must all recognize the serious existential threat from the pandemic, and we must continue to practice IPAC for our safety and that of the others, even when vaccination and chemotherapy have become generally available.
